# Employment and Health Burden Changes Among Medicaid Expansion Enrollees

**DOI:** 10.1001/jamahealthforum.2025.4639

**Published:** 2025-10-31

**Authors:** Minal R. Patel, Sarah J. Clark, Erin Beathard, Matthias Kirch, Nicolas Box, Renuka Tipirneni, John Z. Ayanian, Susan D. Goold

**Affiliations:** 1 Institute for Healthcare Policy and Innovation, University of Michigan, Ann Arbor; 2 University of Michigan School of Public Health, Ann Arbor; 3 Department of Pediatrics, University of Michigan Medical School, Ann Arbor; 4 Department of Internal Medicine, University of Michigan Medical School, Ann Arbor; 5 Gerald R. Ford School of Public Policy, University of Michigan, Ann Arbor

## Abstract

This cohort study evaluates the association between employment status and level of health burden among beneficiaries of a Michigan Medicaid health plan.

## Introduction

Medicaid coverage promotes health and economic well-being for uninsured low-income adults. Medicaid expansion without work requirements has been associated with improved employment, health, and care access.^1,2,3,4^ With Medicaid expansion in Michigan, employment rates increased alongside health improvements among enrollees.^2,5^ We aimed to examine the association between health burden levels and employment outcomes by analyzing employment data over 3 years.

## Methods

This cohort study of 4090 Healthy Michigan Plan (HMP) enrollees used a computer-assisted telephone survey in Arabic, English, and Spanish conducted from January 1 to October 31, 2016 (53.7% response rate), with a first follow-up survey of 3104 respondents (83.4%) from March 2017 to January 2018 and a second follow-up survey of 2608 (89.4%) from June 2018 to January 2019.^4,5,6^ The University of Michigan and Michigan Department of Health and Human Services Institutional Review Boards deemed this study exempt from review; no informed consent was required. We followed the STROBE reporting guideline.

Measures included employment status at each survey, a composite self-reported measure of health burden (overall health status, days of poor physical and mental health, and days that health limited usual activities [eMethods in Supplement 1]^6^), and demographic and clinical characteristics. Survey weights accounted for sample design, nonresponse, and poststratification adjustments. We evaluated the association between health burden and employment using Pearson χ^2^ tests and compared health burden at baseline (2016) vs follow-up using unadjusted mixed-effects logistic regression. We used bivariate analysis to assess the association between health burden changes and employment changes. This association was estimated using multivariable logistic regression, adjusting for age, sex, race and ethnicity, income, region, and chronic conditions (derived from administrative claims). Analyses were conducted in July 2024 with Stata 17 (StataCorp).

## Results

Table 1 shows weighted baseline characteristics for HMP beneficiaries (mean [SD] age, 40.4 [12.8] years; 51.5% female) who completed all surveys. Of these beneficiaries, 57.2% (95% CI, 54.8%-59.6%) had a chronic condition and 48.2% (95% CI, 45.9%-50.4%) were employed. Of those employed in 2018, 67.4% (95% CI, 64.4%-70.2%) were full-time. Of those who gained employment since baseline, 54.6% (95% CI, 48.4%-60.7%) worked full-time.

**Table 1.** Baseline Demographic and Clinical Characteristics of Medicaid Expansion Beneficiaries

Characteristic	No.	Weighted proportion (95% CI)
Age, median (IQR), y^a^		
19-34	736	39.6 (37.2-42.1)
35-50	812	34.5 (32.2-36.8)
51-64	1060	25.9 (24.1-27.8)
Sex^a^		
Male	1004	48.5 (46.1-50.9)
Female	1604	51.5 (49.1-53.9)
Race and ethnicity^b^^,^^c^		
Hispanic	107	4.5 (3.6-5.6)
Non-Hispanic Black	539	26.9 (24.8-29.0)
Non-Hispanic White	1742	58.7 (56.4-60.9)
Non-Hispanic other^d^	186	9.9 (8.4-11.6)
Income, FPL category, %^a^		
0-35	1015	51.8 (50.8-52.9)
36-99	919	28.5 (27.5-29.5)
≥100	674	19.7 (18.8-20.6)
Employed or self-employed^b^		
Yes	1311	48.2 (45.9-50.4)
No	1297	51.8 (49.6-54.1)
State region^a^		
Northern	488	9.0 (8.5-9.4)
Central	822	28.5 (27.5-29.5)
Southern	526	18.5 (17.6-19.4)
Detroit metro	772	44.0 (42.9-45.1)
Health status^b^		
Excellent	219	10.9 (9.4-12.6)
Very good	677	26.1 (24.1-28.3)
Good	912	33.1 (30.9-35.3)
Fair	588	22.6 (20.7-24.7)
Poor	206	7.3 (6.2-8.7)
Health burden^b^		
Substantial	489	18.4 (16.6-20.2)
Moderate	319	12.7 (11.2-14.4)
Minimal	1689	68.9 (66.7-71.1)
Chronic condition^a^^,^^e^		
Yes	1676	57.2 (54.8-59.6)
No	932	42.8 (40.4-45.2)

Abbreviation: FPL, federal poverty level.

^a^ Variable derived from 2016 administrative claims data.

^b^ Variable derived from 2016 enrollee survey.

^c^ Self-reported race was collected as an open-ended question and categorized manually. Race and ethnicity data were included in the study given the well-documented racial and ethnic disparities in employment access and health outcomes that could confound the association between health burden changes and employment outcomes among Medicaid beneficiaries.

^d^ Non-Hispanic other includes multiracial (n = 72), Asian (n = 21), American Indian or Alaska Native (n = 14), Pacific Islander (n = 2), or some other race (n = 77).

^e^ Conditions include diabetes, heart disease, stroke, arthritis (osteoarthritis or rheumatoid arthritis), chronic obstructive pulmonary disease, asthma, hypertension, and cancer.

Employment gains from 2016 to 2018 were observed across health burden levels: 18.9% (95% CI, 15.2%-23.3%) to 32.2% (95% CI, 27.2%-37.5%) for substantial, 43.1% (95% CI, 36.5%-49.8%) to 54.1% (95% CI, 47.3%-60.7%) for moderate, and 57.9% (95% CI, 55.0%-60.7%) to 67.5% (95% CI, 64.7%-70.2%) for minimal. Beneficiaries with substantial or moderate health burden at baseline whose health improved at follow-up demonstrated the largest employment gains (Table 2). Among those with substantial or moderate health burden in 2016 who gained employment, improved health was associated with working full-time (54.1% [95% CI, 41.3%-66.3%] vs 27.5% [95% CI, 13.9%-47.1%]), while no improvement was associated with working part-time (71% [95% CI, 51.6%-84.9%] vs 43.2% [95% CI, 31.1%-56.1%]).

**Table 2.** Beneficiaries Employed at Baseline and Follow-Up by Level of Health Burden in 2016 and Improvement Between 2016 and 2018

Level of health burden	Unweighted No.	Employed beneficiaries, weighted % (95% CI)		
		2016	2017	2018
Substantial				
Got better	240	25.5 (19.4-32.7)	42.0 (34.5-49.9)	46.8 (39.2-54.6)
Stayed the same	236	12.8 (8.8-18.3)	16.3 (11.5-22.5)	18.2 (12.8-25.2)
Moderate				
Got better	159	47.9 (38.5-57.4)	65.5 (56.1-73.9)	67.0 (57.4-75.3)
Got worse	68	34.1 (21.5-49.3)	32.6 (20.6-47.2)	30.1 (18.6-44.8)
Stayed the same	79	41.4 (29.0-55.0)	48.3 (35.2-61.6)	47.2 (34.2-60.6)
Minimal				
Got worse	233	52.3 (44.3-60.2)	59.6 (51.5-67.2)	51.0 (43.1-58.9)
Stayed the same	1414	58.9 (55.7-62.0)	69.4 (66.4-72.3)	70.8 (67.7-73.6)

Among unemployed beneficiaries in 2016 with improved health burden, 20.7% (95% CI, 15.0%-27.8%) achieved full-time and 16.5% (95% CI, 11.4%-23.3%) achieved part-time employment in 2018. Overall, 38.3% (95% CI, 31.1%-46.0%) of unemployed beneficiaries with improved health were employed in 2018 vs 13.5% (95% CI, 9.3%-19.2%) with unchanged or worsened health. Adjusting for demographic characteristics, unemployed beneficiaries with improved health burden were more likely to be employed (adjusted odds ratio [AOR], 4.13; 95% CI, 2.33-7.31) than those with the same or worsened health burden. Health burden improvements were associated with full-time employment gains (AOR, 6.12; 95% CI, 2.66-14.05).

## Discussion

Medicaid expansion beneficiaries reported employment gains across health burden. Among those unemployed in 2016, those with improved health burden were more likely to experience employment gains.

Study limitations include self-reported outcomes, single-state data, and lack of information about whether beneficiaries were seeking work or had other employment barriers. Congress included work requirements in the One Big Beautiful Bill Act for all 40 Medicaid expansion states. As states implement these requirements, they should minimize administrative burden while providing exemptions for people with substantial or moderate health burdens. Furthermore, work requirements need rigorous evaluation to ascertain changes in employment, coverage, and health.

## Notes

Supplement 1.
eMethods

Supplement 2.
Data Sharing Statement
