# Supplementary material for: Employment and Health Burden Changes Among Medicaid Expansion Enrollees
Source: JAMA Health Forum. 2025 Oct 31;6(10):e254639. doi: 10.1001/jamahealthforum.2025.4639 (PMC12579339; doi:10.1001/jamahealthforum.2025.4639)
Supplement: Supplement 1. — eMethods [file jamahealthforum-e254639-s001.pdf]

Supplemental Online Content

Patel MR, Clark SJ, Beathard E, et al. Employment and health burden changes among Medicaid expansion enrollees. *JAMA Health Forum*. 6(10):e254639. doi:10.1001/jamahealthforum.2025.4639

eMethods

This supplemental material has been provided by the authors to give readers additional information about their work.

© 2025 Patel MR et al. *JAMA Health Forum*

eMethods

The information in this Supplement is abstracted from the Healthy Michigan Plan Section 1115 Demonstration Summative Evaluation Report, prepared by the University of Michigan Institute for Healthcare Policy and Innovation for the Michigan Department of Health and Human Services.

Composite measure of health burden

We developed a composite self-reported measure of health burden using the following survey items that were asked at each survey:

- Overall health status: In general, would you say your health is…excellent, very good, good, fair, or poor?
- Days poor physical health: For how many days during the last 30 days was your physical health not good?
- Days poor mental health: For how many days during the last 30 days was your mental health not good?
- Days health limited usual activities: During the last 30 days, for how many days did poor physical or mental health keep you from doing your usual activities?

Based on responses to these items, respondents were classified as having minimal, moderate, or substantial health burden at the time of each of their surveys. Only respondents who answered all items were included in this measure.

Composite health burden definitions

	**Substantial health burden** ≥2 of following:	**Moderate health burden** ≥2 of following:	**Minimal health burden** 0-1 of following:
Overall health status	Poor	Fair or poor	Fair or poor
Days in last 30 with physical health not good	15+ days	7+ days	7+ days
Days in last 30 with mental health not good	15+ days	7+ days	7+ days
Days in last 30 where poor health kept you from doing usual activities	15+ days	7+ days	7+ days

Regression Model estimating the association of changes in health burden with changes in employment

$$\log\left(\frac{P(y_i = 1)}{1 - P(y_i = 1)}\right) = \beta_0 + \beta_1 \boldsymbol{B}_i + \beta^x \boldsymbol{Ind}_i + \epsilon_i$$

where y_i is the dependent variable of interest (new employment gained) for individual i who was unemployed at baseline; $\boldsymbol{B}_i$ is a dummy variable on the individual level representing any improvement in burden; $\mathbf{Ind}_i$ is a vector of individual controls (age, gender, race and ethnicity, fpl level, region, and chronic conditions).

© 2025 Patel MR et al. *JAMA Health Forum*
