# Supplementary material for: Employment and Health Burden Changes Among Medicaid Expansion Enrollees
Source: JAMA Health Forum. 2025 Oct 31;6(10):e254639. doi: 10.1001/jamahealthforum.2025.4639 (PMC12579339; doi:10.1001/jamahealthforum.2025.4639)
Supplement: Supplement 2. — Data Sharing Statement [file jamahealthforum-e254639-s002.pdf]

Data Sharing Statement

Patel. Employment and Health Burden Changes Among Medicaid Expansion Enrollees. *JAMA Health Forum*. Published October 31, 2025. doi:10.1001/jamahealthforum.2025.4639

Data
Data available: No

Additional Information
Explanation for why data not available: Data will be made available upon request
